# Electrical alternans vs. pseudoelectrical alternans

**DOI:** 10.3402/jchimp.v2i1.17610

**Published:** 2012-04-30

**Authors:** Marc Mugmon

**Affiliations:** Department of Medicine, Union Memorial Hospital, Baltimore, MD, USA

## Abstract

Beat-to-beat variations in QRS amplitude or axis may be caused by several conditions.

A 70-year-old female was admitted with a two-month history of rectal bleeding. Subsequent evaluation revealed moderately differentiated carcinoma of the rectum. Metastatic workup revealed no metastases. Chest CT demonstrated a possible small anterior pericardial effusion versus pericardial thickening, but no echocardiogram was obtained. Excision of the mass was undertaken and a colostomy performed. Electrocardiography (Fig. 1) revealed a beat-to-beat shift in the QRS axis and amplitude. Electrical alternans is characterized by such variation in either axis or QRS amplitude. The patient's symptoms were related only to her rectal mass and consequent bleeding and anemia. Electrolytes were normal and she denied dyspnea, chest pain or palpitations.

**Fig. 1:**
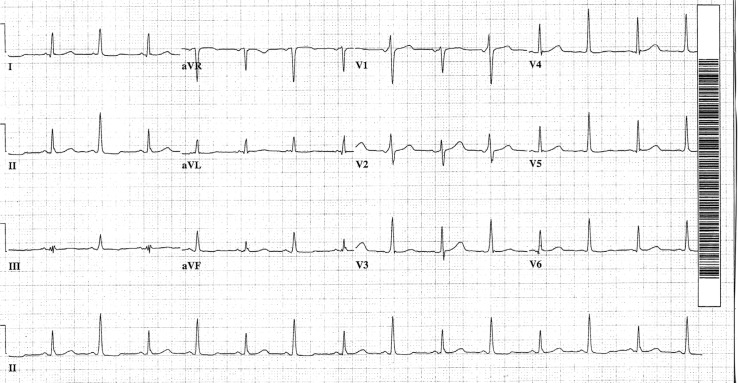
Alternating QRS amplitude and axis.

A typical cause of electrical alternans is a pericardial effusion, and is due to periodic wobbling of the heart in the pericardium (1). However, alternating axis shift may be due not to mechanical shifting of the heart, but to alternating conduction abnormality, such as intermittent fascicular or bundle branch block. In 1978, Klein, Segni and Kaplinsky coined the term ‘pseudoelectrical alternans’ in a case report of intermittent left anterior hemiblock, in which the axis shifted every other beat due to the development of alternating normal and then leftward axis shift, presumably related to procainamide therapy (2).

Whenever what appears to be electrical alternans is not due to a large pericardial effusion, then pseudoelectrical alternans should be considered. Pseudoelectrical alternans is due to alternation in axis or amplitude because of events that alter conduction and do not alter the physical orientation of the heart.

Two possibilities in the patient described above include ventricular bigeminy and intermittent pre-excitation. In ventricular bigeminy, fusion beats are generated due to the timing of the ventricular extrasystoles, which fuses with the normal QRS, causing a shift in the axis with every other beat. Intermittent ventricular pre-excitation due to an accessory pathway could also cause an alternating shift in axis. Fig. 1 does suggest the presence of a delta wave and a short PR interval in lead V2, although this configuration may also occur with a fusion beat.

Since the mechanism of the alternation in QRS magnitude in the patient described above does not appear to be due to shifting of the heart, (no large pericardial effusion was present) then the term pseudoelectrical alternans would be more accurate in this patient.
